# Modeling the Potential Distribution of the Malaria Vector *Anopheles (Ano.) pseudopunctipennis* Theobald (Diptera: Culicidae) in Arid Regions of Northern Chile

**DOI:** 10.3389/fpubh.2021.611152

**Published:** 2021-05-11

**Authors:** Lara Valderrama, Salvador Ayala, Carolina Reyes, Christian R. González

**Affiliations:** ^1^Laboratorio de Entomología, Subdepartamento de Genética Molecular, Instituto de Salud Pública de Chile, Santiago, Chile; ^2^Programa de Magíster en Ciencias mención Entomología, Universidad Metropolitana de Ciencias de la Educación, Santiago, Chile; ^3^Departamento de Asuntos Científicos, Instituto de Salud Pública de Chile, Santiago, Chile; ^4^Instituto de Entomología, Universidad Metropolitana de Ciencias de la Educación, Facultad de Ciencias Básicas, Santiago, Chile

**Keywords:** species distribution model, climate change, maxent, malaria, Latin America, One health

## Abstract

The extreme north of Chile presents a subtropical climate permissive of the establishment of potential disease vectors. *Anopheles* (*Ano*.) *pseudopunctipennis* is distributed from the south of the United States to the north of Argentina and Chile, and is one of the main vectors of malaria in Latin America. Malaria was eradicated from Chile in 1945. Nevertheless, the vector persists in river ravines of the Arica and Tarapacá regions. The principal effect of climate change in the north of Chile is temperature increase. Precipitation prediction is not accurate for this region because records were erratic during the last century. The objective of this study was to estimate the current and the projected distribution pattern of this species in Chile, given the potential impact due to climate change. We compiled distributional data for *An*. (*Ano*.) *pseudopunctipennis* and constructed species distribution models to predict the spatial distribution of this species using the MaxEnt algorithm with current and RCP 4.5 and 8.5 scenarios, using environmental and topographic layers. Our models estimated that the current expected range of *An*. (*Ano*.) *pseudopunctipennis* extends continuously from Arica to the north of Antofagasta region. Furthermore, the RCP 4.5 and 8.5 projected scenarios suggested that the range of distribution of *An. (Ano.) pseudopunctipennis* may increase in longitude, latitude, and altitude limits, enhancing the local extension area by 38 and 101%, respectively, and local presence probability (>0.7), from the northern limit in Arica y Parinacota region (18°S) to the northern Antofagasta region (23°S). This study contributes to geographic and ecologic knowledge about this species in Chile, as it represents the first local study of *An*. (*Ano*.) *pseudopunctipennis*. The information generated in this study can be used to inform decision making regarding vector control and surveillance programs of Latin America. These kinds of studies are very relevant to generate human, animal, and environmental health knowledge contributing to the “One Health” concept.

## Introduction

The extreme north of Chile presents a subtropical climate, permissive of the establishment of several mosquito species. Some of these species are vectors of different pathogens affecting humans, like *Aedes* (*Ste*.) *aegypti, Anopheles* (*Ano*.) *pseudopunctipennis*, and *Culex* (*Cux*.) *quinquefasciatus*. *An*. (*Ano*.) *pseudopunctipennis* Theobald is distributed from the south of the United States (40°N) to the north of Argentina (30°S) and Chile along the Andes, and it extends to Venezuela and the Lesser Antilles (1, 2). It is found at altitudes from sea level up to 3,200 m. Females oviposit in river pools in mountain areas, and larvae are tolerant to wide temperature ranges and droughts because rainfall may destroy their breeding sites. Nevertheless, natural pools, rice plantations, and wetlands can also support *An*. (*Ano*.) *pseudopunctipennis*, when located in proximity to human populations (1–4). Forty-one species of *Anopheles* have been described as vectors of malaria (5), and *An*. (*Ano*.) *pseudopunctipennis* is an important vector of malaria in different countries of South America [5; (4)]. Malaria was endemic in northern Chile until 1945, the year in which the last autochthonous case of malaria was reported. However, the vector is still present, confined to natural breeding sites in riversides in Lluta Valley, Quebrada Vítor, Camarones, and Tarapacá Valley ravines in rural areas of the north of Chile [unpublished data, Laboratorio de Referencia de Entomología ISP; (6)]. Although there is no local transmission of malaria in Chile, there are, on average, 12 imported registered cases per year in the last two decades (7–9). Furthermore, there are several recent records of *An*. (*Ano*.) *pseudopunctipennis* near urban areas in Arica (18°48′33^′′^S latitude, 70°33′33^′′^O longitude) and Matilla (20°51′42^′′^S latitude, 69°36′14^′′^O longitude) [unpublished data, Unidad de Emergencias y Desastres, SEREMI de Arica y Parinacota; unpublished data, Laboratorio de Referencia de Entomología ISP; (10)]. These factors support the risk of malaria reintroduction, particularly given that the north of Chile is considered an area of immigration from the malaria endemic countries of Perú and Bolivia (11).

The north of Chile is one of the most arid regions in the world, and it is characterized as being exposed to intense solar radiation and comprised of territories at different altitudes (12). There is no consensus about climate change predictions in the north of Chile, especially regarding precipitation because record keeping has been erratic over the last century (12). Nevertheless, temperature is expected to increase by 1°C on the coast and by 4°C in the Andes mountains in the “Norte Grande” region (13). However, precipitation may decrease slightly in the north of Chile, especially in the Andean plateau (12, 14, 15), even though rainfall could increase in the Andean foothills (12). Temperature and precipitation changes and topographic characteristics of the terrain may impact the potential range of *An*. (*Ano*.) *pseudopunctipennis*, as demonstrated for other anopheline species (16–18). Important knowledge gaps remain regarding the potential effects of climate and climate change on the emergence of several vector-borne diseases in the world. Here, we provide detailed local maps of the current expected geographical range of *An*. (*Ano*.) *pseudopunctipenni*s in Chile and examine possible changes in the potential distribution of this species under future climatic conditions, based on outputs of 10 global climatic models and two representative concentration pathways (RCP 4.5 and 8.5).

## Materials and Methods

### Data Collection and Study Area

**Figure 1:**
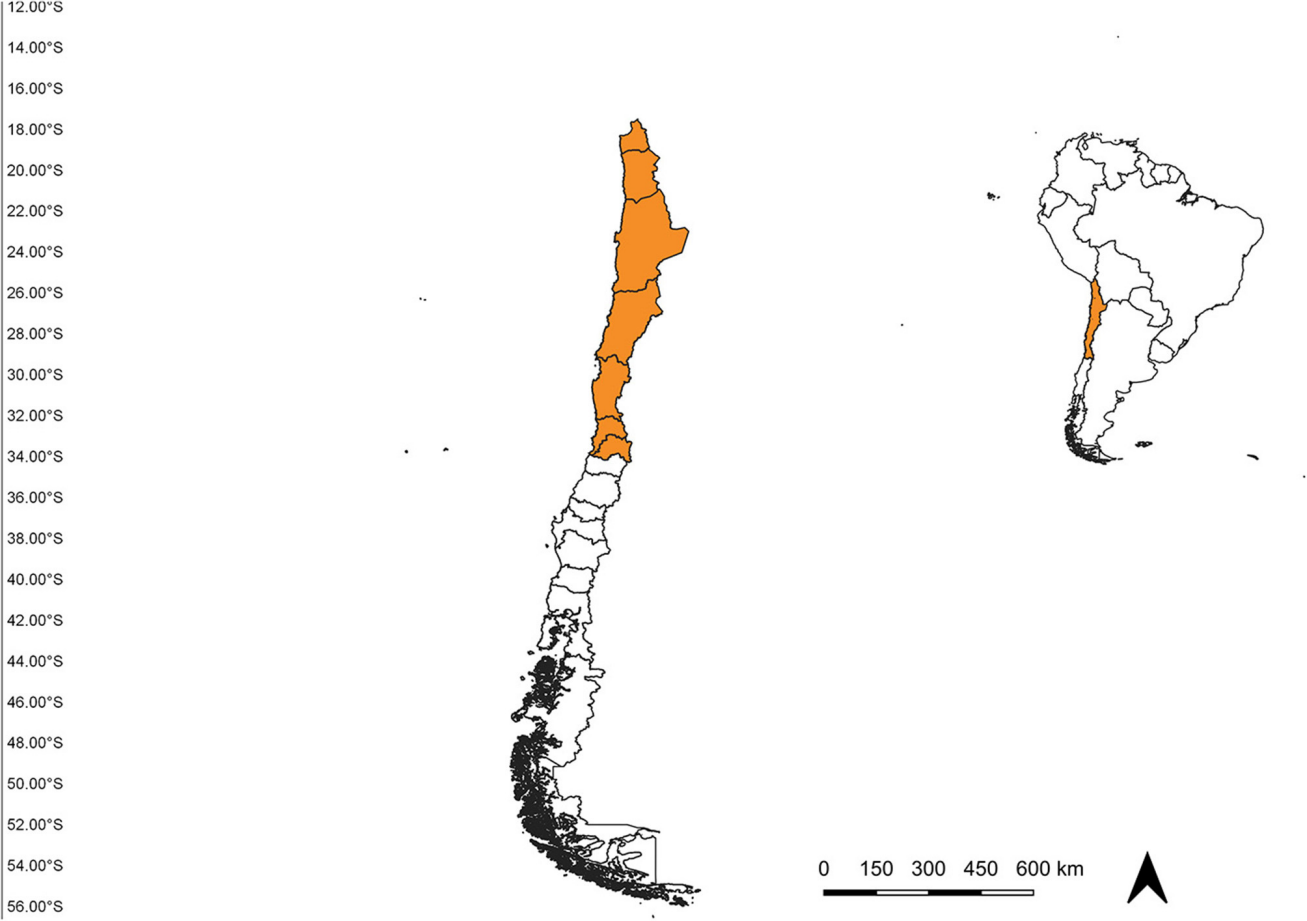
Study area spanning from the Arica region in the north to the Metropolitan region in the south, Chile.

**Figure 2:**
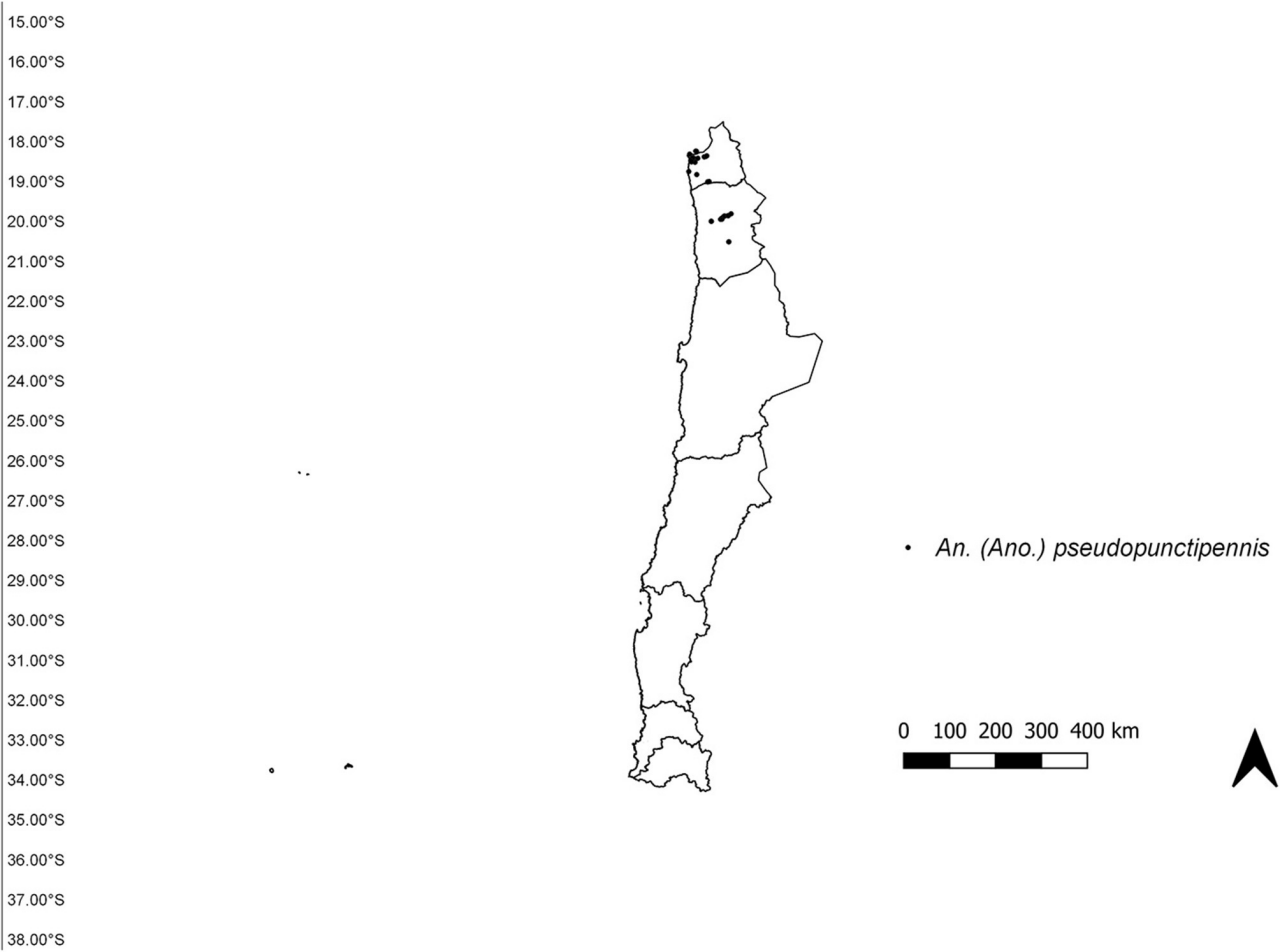
Occurrence records of *Anopheles (Ano.) pseudopuntipennis* in Chile.

The study area was established from the extreme north of Chile (18°S) to the Metropolitan region (34°S) (Figure 1), according to the “Programa de vigilancia vectorial de culícidos del Ministerio de Salud de Chile.” Occurrence data for *An*. (*Ano*.) *pseudopunctipennis* from 2009 until January 2020 were obtained from the “Programa de vigilancia vectorial de culícidos del Ministerio de Salud de Chile” “Laboratorio de Referencia de Entomología del Instituto de Salud Pública de Chile” and Cancino (10). We included all records with geographic coordinates, and filtered data to eliminate duplicate records and those with < 1 km of distance between them (19). A total of 50 records were compiled: 22 from Arica y Parinacota region and 38 from Tarapacá region (Figure 2).

### Climatic Data, Layers, and Projected Models

Data from WorldClim (20) were used to characterize current local climates, using “raster” package in R software, including 15 bioclimatic variables. Bioclimatic variables 8–9 and 18–19 were omitted from the analysis because their validity is questioned (21–23). A layer representing river proximity [given that *An*. (*Ano*.) *pseudopunctipennis* breeding sites are close to streams] and five topographic layers (altitude, slope, exposition, orientation, and flow direction), obtained from “Digital Elevation Model” (DEM) using the “elevatr” package in R software, were included to estimate the “topographic roughness index” (TRI), “topographic position index” (TPI), and “topographic wetness index” (TWI). However, we had to eliminate the Loa river from this variable because its water is brackish (24). We included another layer (human footprint) (25) because this species is a malaria vector. All variables were discharged to 1 km^2^ resolution (30 s). For the projected models, we used two different climate change scenarios: RCP 4.5 and RCP 8.5. These were based on 10 global climate models covering the period from 2061 to 2080 from: ACCESS1-0 (AC) (Australian Community Climate and Earth-System Simulator), BCC-CSM1-1 (BC) (Beijing Climate Center and China Meteorological Administration), CCSM4 (CC) (National Center for Atmospheric Research), CNRM-CMIP5 (CN) (Centre National de Recherches Météorologiques and Centre Européen de Recherche et Formation Avancée en Calcul Scientifique), GFDL-CM3 (GF) (Geophysical Fluid Dynamics Laboratory), HadGEM2-ES (HE) (National Institute of Meteorological Research/Korea Meteorological Administration), INMCM4 (IN) (Institute of Numerical Mathematics Climate Model), IPSL-CMSA-LR (IP) (Institut Pierre-Simon Laplace), MIROC5 (MC) (Atmosphere and Ocean Research Institute, National Institute for Environmental Studies and Japan Agency for Marine-Earth Science, and Technology), and NorESM1-M (NO) (Norwegian Climate Centre) to 2061–2080 period of global climate models.

### Species Distribution Modeling

We applied a correlation analysis and selected variables based on the variance inflation factor (VIF < 10) to avoid an over-adjustment in the models (17). We made a current species distribution model using the MaxEnt algorithm (Maxent v.3.4.1) (20) with 50 replicates (26) and 3,600 pseudoabsences (27). We selected the minimum number of variables, based on *Jackknife test*, response curve of each variable graphic and “area under the curve” value (AUC > 0.9) (18, 26, 28–30). Then, we conducted a logistic regression to explain the relation of each one of the variables with *An*. (*Ano*.) *pseudopunctipennis* presence probability (31).

### Evaluation of Species Distribution Models

We selected metrics parameters [regularization multiple (RM) and function type: linear, product, quadratic, hinge, and threshold] based on the lowest “Akaike Information Criterion corrected” value (AICc) (28, 32–34) of ENMeval evaluation (35) in R software (36). We applied metrics parameters selected in the MaxEnt algorithm for current and projected conditions. We chose the best model based on the prevalence approach, average probability/suitability, sensitivity-specificity sum maximization approach, the sensitivity-specificity equality approach, and AUC value (26, 37).

### Presence Probability Extension Area Calculation

We calculated the current and projected presence probability extension area based on the “maximum training sensitivity plus specificity logistic threshold.”

## Results

According to the ENMeval evaluation, the metrics parameters selected to apply to the Maxent approach were RM = 1.5 and linear, quadratic, and product functions.

Furthermore, we chose the GF model as the best model due to prevalence approach, average probability/suitability, sensitivity-specificity sum maximization approach, sensitivity-specificity equality approach, and AUC value (Table 1).

**Table 1:** Evaluation results to select the best model to project.

**RCP**	**Model**	**Sensitivity = specificity**	**Sensitivity-specificity sum maximization**	**Predicted prevalence = observed prevalence**	**Predicted**	**Average probability**	**AUC**
4.5	AC	0.435	0.415	0.71	0.0228	0.023	0.9962
4.5	BC	0.385	0.365	0.67	0.0201	0.0202	0.9958
4.5	CC	0.4	0.39	0.66	0.0209	0.0215	0.996
4.5	CN	0.38	0.355	0.7	0.022	0.0211	0.9953
4.5	GF	0.49	0.465	0.79	0.0256	0.0251	0.9946
4.5	HE	0.475	0.46	0.73	0.0236	0.0234	0.9961
4.5	IN	0.375	0.345	0.69	0.0214	0.0208	0.9957
4.5	IP	0.455	0.44	0.74	0.0236	0.0239	0.996
4.5	MC	0.44	0.43	0.72	0.0228	0.0221	0.9962
4.5	NO	0.43	0.43	0.72	0.0228	0.0223	0.9958
8.5	AC	0.49	0.48	0.75	0.0245	0.026	0.996
8.5	BC	0.49	0.48	0.76	0.0245	0.0251	0.9956
8.5	CC	0.475	0.46	0.73	0.0237	0.0247	0.9961
8.5	CN	0.495	0.48	0.76	0.0245	0.0243	0.9958
8.5	GF	0.69	0.68	0.86	0.0294	0.0355	0.9959
8.5	HE	0.575	0.555	0.79	0.027	0.0282	0.9965
8.5	IN	0.485	0.47	0.75	0.0247	0.0247	0.9958
8.5	IP	0.55	0.54	0.83	0.0275	0.0295	0.9957
8.5	MC	0.51	0.485	0.77	0.0264	0.0254	0.9956
8.5	NO	0.49	0.48	0.74	0.0245	0.0235	0.9955

**Figure 3:**
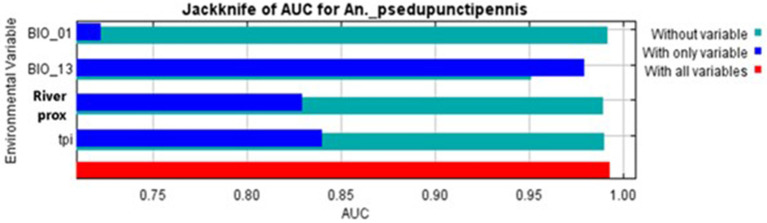
*Jackknife* test model with variables BIO1, Bio13, river proximity, and topographic position index (TPI).

According to the *Jackknife* test, the best predictor variable for the distribution of *An*. (*Ano*.) *pseudopunctipennis* in Chile was “precipitation during the wettest month” (BIO13), second was “topographic position index” (TPI), third was “river proximity,” and fourth was “annual mean temperature” (BIO1) (Figure 3).

Logistic regression results indicated that “BIO1” and “BIO13” were positively related to the model, while “TPI” and “river proximity” were negatively related to it. Nevertheless, “BIO13” was not significant to the model (*p* = 0.752) (Table 2).

**Table 2:** Regression logistic results using variables BIO 1, BiO 13, TPI, and river proximity.

**Variable**	**ß coefficient**	**Standard error**	***p*-value**
BIO1	0.001898	0.000344	<0.01
BIO13	0.00001278	0.00004038	0.752
TPI	−0.0003896	0.00005867	<0.01
River proximity	−0.000000084298	0.000000007712	<0.01

**Figure 4:**
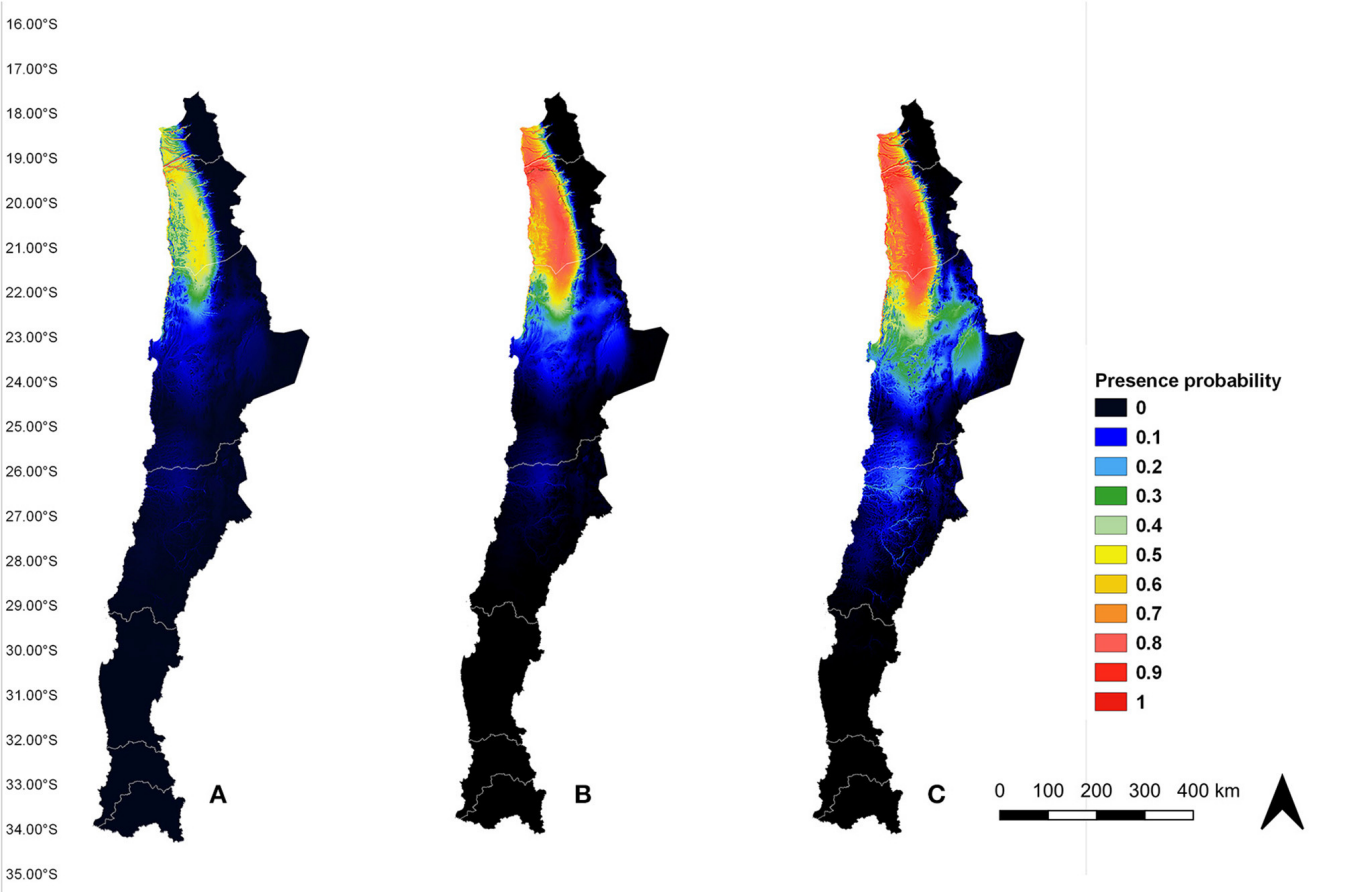
*An. (Ano.) pseudopunctipennis* potential distribution model in Chile under current **(A)**, RCP 4.5 **(B)**, and RCP 8.5 **(C)** scenarios. Model GF.

All models demonstrated to have an AUC value up to 0.9, proving they have an excellent predictive performance. The current potential distribution of *An*. (*Ano*.) *pseudopunctipennis* in Chile (Figure 4A) showed there is a high probability of presence (orange and red areas) in several river ravines of Arica y Parinacota and Tarapacá regions, between 18°21′S and 19°37′S latitudes. This result validated the model because high presence probability areas matched with the records used for the analysis. Also, medium presence probability (green and yellow areas) extended from the north of Arica y Parinacota region (18°21′S latitude) to the north of Antofagasta region (22°28′S latitude). Low presence probability (blue area) extended from the north of Antofagasta region (21°53′S latitude) to the center of the same region (24°40′S latitude). In the future scenarios (Figures 4B,C), the areas of high *An*. (*Ano*.) *pseudopunctipennis* presence probability (orange and red areas) overlapped with the medium presence probability area in the current model, thus extending the zone of high presence probability from the north of Arica y Parinacota region to the north of Antofagasta region. There was a low presence probability (blue area) in “Salar de Atacama” (23°30′S latitude, 68°15′O longitude) in the current (Figure 4A) and RCP 4.5 scenario (Figure 4B), but this area increased its presence probability to medium (green area) in the RCP 8.5 scenario (Figure 4C). Furthermore, there is a low presence probability (blue and light blue areas) in the river ravine situated at the border of the Antofagasta region and Atacama region (between 25°23′S and 26°40′S latitudes) (Figures 4B,C). Comparing the presence probability extension areas in the three scenarios (current, RCP 4.5, and RCP 8.5), it increased to 38% (from 39.353 to 54.378 km^2^) in the RCP 4.5 scenario, and it increased to 101% (from 39.353 to 79.299 km^2^) in the RCP 8.5 model, according to the “maximum training sensitivity plus specificity logistic threshold” (0.2652).

## Discussion

This study assembled a local data set summarizing occurrences of *An*. (*Ano*.) *pseudopunctipennis* and provided detailed maps of its potential geographic distribution under current and future climatic conditions. The latter objective is important in anticipating any possible future distributional changes of *An*. (*Ano*.) *pseudopunctipennis* and the potential risk to human health posed by the reintroduction and transmission of malaria in northern Chile.

Under both climatic change scenarios, it was probable that conditions would permit an expansion of the geographic range of *Anopheles* species in several world regions (38). According to the *An*. (*Ano*.) *pseudopunctipennis* distribution model in Chile, its presence probability would increase in its geographic extent under both scenarios (by 38% in RCP 4.5 and by 101% in RCP 8.5). These results fitted with the expected projection for the geographic range of *An*. (*Ano*.) *pseudopunctipennis* given insect adaptation to climate change effects (39–41).

In the three scenarios analyzed (the current scenario, RCP 4.5, and RCP 8.5), we observed an area of medium presence probability for the current model (Figure 4A) and of high presence probability in the RCP 4.5 and RCP 8.5 models (Figures 4B,C), extending from the north of the Arica y Parinacota region (18°21′S latitude) to the north of the Antofagasta region (21°53′S latitude), along the Andes mountains. This area shares similar topology, bioclimatic, and vegetational characteristics, typical of the desert (42).

“TPI” and “river proximity” were the topographic layers most relevant in the model. Both variables were negatively related to the model, meaning there was a higher *An*. (*Ano*.) *pseudopunctipennis* presence probability in areas capable of retaining the water from river flooding. This reflects the preference of this species to lay eggs in fresh water pools formed near riverbanks (4).

The projections for the north of Chile should be interpreted with caution due to inconsistent record keeping regarding precipitation during the last century. In addition, northern Chile precipitation records are lower than what is typical in tropical areas, probably causing errors in statistical models (12). Nevertheless, precipitation level is a relevant variable to predict the presence of this species because it is important for the aquatic development of immature mosquito stages, especially in arid regions like northern Chile. Furthermore, higher precipitation is associated with an increased reproductive rate and distribution expansion in insects (39–41). The *An*. (*Ano*.) *pseudopunctipennis* population in Chile is already known to increase after summer rainfall due to a plateau-style winter (“Invierno altiplánico”). It is not possible to predict how this climate phenomenon will behave under climate change scenarios because it depends on the “South Pacific Anticyclone” (42). However, an intensification of this type of phenomenon has been observed in association with the effects of climate change in recent years (43). Thus, there is a possibility that summer rains will increase in the RCP 4.5 and 8.5 scenarios, affecting positively the presence probability of *An*. (*Ano*.) *pseudopunctipenni*s. Nevertheless, precipitation layers are controversial for this vector distribution model because intense rains are also responsible for the destruction of its breeding sites. Although “precipitation in the wettest month” (BIO13) was the most relevant variable according to the *Jackknife* test, logistic regression showed that “BIO13” was not significant for the model (*p* = 0.752), perhaps because of its controversial contribution to *An*. (*Ano*.) *pseudopunctipennis* presence.

“Annual mean temperature” (BIO1) increases also had an impact in the distribution of this vector. Higher temperatures decrease the duration of the development cycle and increase fecundity, survival rates, population density, and dispersion capacity in insects (39–41). Therefore, increases in projected temperatures under both climate change scenarios would be expected to enhance the geographic extent of the presence probability area.

“Human footprint” was not a predictive variable for the model because this vector is distributed in rural areas, removed from human population centers in northern Chile (6), and feeding primarily on local animal hosts (44, 45).

Under both projected scenarios, there was a low (Figure 4B) to medium (Figure 4C) presence probability of this species in the “Salar de Atacama.” As in the case of the Loa river, the salinity of the water in the “Salar de Atacama” would not permit the development of *An. (Ano.) pseudopunctipennis* immature stages (24).

Under all three scenarios, there was a low presence probability of this vector around the border of the Antofagasta and Atacama regions (between 25°23′S and 26°40′S latitudes). Although topographic and bioclimatic characteristics are likely similar to the areas where *An. (Ano.) pseudopunctipennis* is present, it would be unusual to find this species in this region because it is completely isolated from the areas with known presence of this vector.

Nevertheless, several *An. (Ano.) pseudopunctipennis* specimens have been collected in Arica city and the nearby Matilla village (Tarapacá region) in the last 3 years [unpublished data, Laboratorio de Referencia de Entomología ISP; (10)]. Perhaps this species is re-infesting areas where it was historically found, or perhaps it is adapting to new climatic conditions, like temperature increment and intense precipitations events. This situation poses a risk of re-introducing autochthonous malaria transmission to Chile, especially because northern Chile is a transit and immigration zone for people coming from malaria-endemic countries, like Perú and Bolivia (11).

In conclusion, these analyses provide guidance regarding areas that are potentially vulnerable to the reintroduction of autochthonous malaria transmission in Chile and will help to optimize the response to any eventual outbreaks of the disease. Species distribution models are very relevant to generate human, animal, and environmental health knowledge contributing to the “One Health” concept.

## Data Availability Statement

Publicly available datasets were analyzed in this study. This data can be found at: https://www.portaltransparencia.cl/PortalPdT/ingreso-sai-v2?idOrgTa=AO005.

## Author Contributions

LV designed the study, made the data analysis, and wrote the manuscript. SA helped with the data analyses and revised the manuscript. CR made the field work and revised the manuscript. CRG collaborated in the study design, made the field work, and revised the manuscript. All authors contributed to the article and approved the submitted version.

## Conflict of Interest

The authors declare that the research was conducted in the absence of any commercial or financial relationships that could be construed as a potential conflict of interest.
